# The occurrence of adverse events in low-risk non-survivors in pediatric intensive care patients: an exploratory study

**DOI:** 10.1007/s00431-018-3194-y

**Published:** 2018-06-26

**Authors:** Carin W. Verlaat, Cynthia van der Starre, Jan A. Hazelzet, Dick Tibboel, Johannes van der Hoeven, Joris Lemson, Marieke Zegers

**Affiliations:** 1 Department of Intensive Care, Radboud Institute for Health Sciences, Radboud University Medical Center, P.O. Box 9101, Internal Post 709, 6500 HB Nijmegen, The Netherlands; 2 Department of Neonatal and Pediatric Intensive Care, Erasmus University Medical Center–Sophia Children’s Hospital, Rotterdam, The Netherlands; 3 Department of Public Health, Erasmus University Medical Center–Sophia Children’s Hospital, Rotterdam, The Netherlands; 4 Department of Pediatric Intensive Care, Erasmus Medical Center–Sophia Children’s Hospital, Rotterdam, The Netherlands; 5 Department of Intensive Care and IQ Healthcare, Radboud Institute for Health Sciences, Radboud University Medical Center, Nijmegen, The Netherlands

**Keywords:** Adverse events, Complications, Patient safety, Hospital, (P ediatric) intensive care, Trigger tool, Health care quality, Outcome

## Abstract

We studied the occurrence of adverse events (AEs) in low-risk non-survivors (LNs), compared to low-risk survivors (LSs), high-risk non-survivors (HNs), and high-risk survivors (HSs) in two pediatric intensive care units (PICUs). The study was performed as a retrospective patient record review study, using a PICU-trigger tool. A random sample of 48 PICU patients (0–18 years) was chosen, stratified into four subgroups of 12 patients: LNs, LSs, HNs, and HSs. Primary outcome was the occurrence of AEs. The severity, preventability, and nature of the indentified AEs were determined. In total, 45 AEs were found in 20 patients. The occurrence of AEs in the LN group was significantly higher compared to that in the LS group and HN group (AE occurrence: LN 10/12 patients, LS 1/12 patients; HN 2/12 patients; HS 7/12 patients; LN-LS difference, *p* < 0.001; LN-HN difference, *p* < 0.01). The AE rate in the LN group was significantly higher compared to that in the LS and HN groups (median [IQR]: LN 0.12 [0.07–0.29], LS 0 [0–0], HN 0 [0–0], and HS 0.03 [0.0–0.17] AE/PICU day; LN-LS difference, *p* < 0.001; LN-HN difference, *p* < 0.01). The distribution of the AEs among the four groups was as follows: 25 AEs (LN), 2 AEs (LS), 8 AEs (HN), and 10 AEs (HS). Fifteen of forty-five AEs were preventable. In 2/12 LN patients, death occurred after a preventable AE.

*Conclusion*: The occurrence of AEs in LNs was higher compared to that in LSs and HNs. Some AEs were severe and preventable and contributed to mortality.

**Table:** 

**What is Known:**	
*• 59–76% of all PICU patients encounter at least one adverse event during their PICU stay.*	
*• It is unknown if adverse events play a role in death of low-risk PICU patients.*	
**What is New:**	
*• In low-risk PICU non-survivors, occurrence of adverse events is higher compared to low-risk PICU survivors and to high-risk PICU non-survivors.*	
*• Severe and preventable adverse events occur in low-risk PICU non-survivors, some contributing to mortality.*

**Electronic supplementary material:**

The online version of this article (10.1007/s00431-018-3194-y) contains supplementary material, which is available to authorized users.

## Introduction

The mortality rate in the pediatric intensive care unit (PICU) in economically developed countries has decreased in the last decades to approximately 3% [26]. Moreover, a substantial part of the PICU population (55% in a recent study) has a mortality risk of < 1% [35]. Although these are low-risk patients, some of these patients die on the PICU. Patient factors like complex chronic conditions (CCCs) do not explain all deceased patients in this patient group [6, 35]. For quality purposes, it is interesting to analyze whether adverse events (AEs) or even medical errors play a role in the death of low-risk PICU patients [3, 16]. An AE is an unintended injury that results in temporary or permanent disability, death, or prolonged hospital stay and that is caused by health care management rather than by the patient’s underlying disease process [38]. A national project on preventable deaths in Dutch hospitals showed that preventable AEs contributed to 4.1% of hospital deaths [38, 39]. In most international AE studies, (young) children were excluded or the number of included PICU admissions was not specified or very low, so data about PICU patients are scarce [2–4, 18, 38].

Because of their vulnerability, intensive care patients are more prone to iatrogenic events [10, 12, 13]. The incidence of AEs in the PICU population depends on the method used to detect AEs [1, 17, 19, 22, 28, 31, 33, 36]. Studies using a trigger tool method show that 59–76% of all PICU patients encounter at least one AE during their stay [1, 17, 36].

Although one could speculate that AE incidence is higher in the more complex and sicker patients needing extensive support (high-risk patients), AEs also occur in the less severely ill PICU patients [1, 17, 22]. To our knowledge, no studies have focused on the occurrence of AEs in low-risk PICU patients. The incidence of AEs among low-risk patients might be underestimated when only the general PICU population is examined. Analyzing medical records from non-survivors with a low risk of dying is an efficient tool to discover problems in the quality of care [14]. If low-risk PICU patients deteriorate or die because of preventable AEs, there is a potential for improving their outcome.

The aim of this exploratory study was to study the occurrence of AEs in the low-risk non-survivors (LNs), compared to low-risk survivors (LSs), high-risk non-survivors (HNs), and high-risk survivors (HSs) in two PICUs. Of all AEs, we studied the severity, preventability, and nature. The study was designed as a retrospective exploratory study that used chart review to examine the feasibility of detecting AEs in this patient group.

## Methods

### Study design and setting

This is a retrospective patient record study to measure the occurrence of AEs in low-risk non-survivors and to compare the results with patients with a different risk profile and different outcomes, using a random stratified sample of 48 records. The study was performed in two PICUs. Data collection was performed in 2015.

### Admission selection

Admissions in each PICU between 1 January 2006 and 1 January 2012 were stratified into four groups with different risk profiles and different outcomes. The study group consisted of LNs. Three control groups were chosen: LSs, HNs, and HSs. Low-risk admissions were defined as admissions with a mortality risk in the simply recalibrated Pediatric Index of Mortality (PIM) 2 score and/or recalibrated Pediatric Risk of Mortality II score (further referred as “PRISM”) of < 1% [24, 25, 27, 29, 35]. High-risk admissions were defined as admissions with a mortality risk in the simply recalibrated PIM2 and/or PRISM of ≥ 30% [35].

Other inclusion criteria were the following: age < 18 years and PICU length of stay of at least 2 h. Exclusion criteria were the following: patients already deceased before admission (*for example, brain dead patients, admitted for organ donation*), corrected age < 36 weeks (gestational age), invalid or impossible PIM2/PRISM score, and no clinical data available.

The mortality risk scores and PICU outcome data were provided by the national PICU registry (Pediatric Intensive Care Evaluation (PICE) registry) [23]. The PICE registry is a national database containing anonymized information of admission characteristics, severity of illness, and patient outcome. Data quality is assessed using standard procedures including audit site visits. Of all patients, both PIM2 and PRISM scores are collected. The models were recalibrated for the study period to predict the overall mortality in the total population in this period without altering the relative weights of risk factors in the models and thus retaining the discriminative power of the models [35, 37]. A local copy from the PICE registry was sent to the local PICUs including all admissions between 2006 and 2012. The database of these two PICUs (total of 11,216 admissions: PICU-1, 8438 admissions; PICU-2, 2778 admissions) contained 39 LNs.

Since the study was designed as an exploratory study, a selection of roughly one third of the LN was used for the study. Twelve LNs were selected for the study. Because the number of patients between the two participating centers was unequal, nine admissions from PICU-1 and three admissions from PICU-2 were selected for each study group, using a computer-based research randomizer [34]. To avoid different population characteristics, the patients in the control groups (LS (*n* = 12), HN (*n* = 12), HS (*n* = 12)) were stratified based on PICU center, gender, and age category. After stratification, the patients were randomly chosen using the computer-based research randomizer.

To verify if the risk profile of patients was correct, the PIM2 and PRISM scores were checked using available physiologic and laboratory data. If a discrepancy was discovered, e.g., the corrected mortality risk turned out to be >2% in LN and LS or < 30% in HN and HS, the patient was excluded from the study. The next from the list of available patients (with the same risk group/outcome/PICU center/gender/age category) was selected until, in each group, 12 patients were included.

### Data collection

An established set of triggers was modified to local characteristics of the PICU population and was used in a retrospective chart review to discover AEs (Table 4, online only) [1]. In the first stage, patient charts were manually reviewed for the presence of 19 triggers. In the second stage, each positive trigger was followed by an in-depth investigation for the presence of associated AEs. Both stages were performed by a pediatric intensivist (CV) with more than 15 years of PICU experience who was trained in the use of the trigger tool method.

Primary outcomes were the occurrence of AEs and AE rate (AE/PICU day). For the AE rate, only AEs occurring during the PICU admission were included. AEs that occurred shortly before PICU admission and were beyond doubt related to the PICU admission were scored as “AE pre PICU.” The severity of AEs was rated using the National Coordinating Council for Medication Error Reporting and Prevention (NCC MERP) Index for Categorizing Errors (Table 5, online only) [20]. Preventability of AEs was scored on a 6-point scale (Table 6, online only) [3]. AEs with a preventability score of 4–6 were defined as preventable. A preventable AE results from an error in management due to failure to follow accepted practice at an individual or system level. Accepted practice was taken to be “the current level of expected performance for the average practitioner or system that manages the condition in question” [38]. AEs were grouped into eight categories, based on the classification made by Hogan et al. (Table 7, online only) [15]. If problems were encountered in AE determination and categorizing AEs, a decision was taken after discussion within the research group.

The ANZPIC registry diagnostic code list was used for diagnosis classification [30]. An admission was classified as having a CCC or a non-complex chronic condition (NCCC) if either the primary diagnosis, the primary underlying diagnosis, or the first additional diagnosis was a diagnosis defined as a CCC or NCCC according to a modified Feudtner’s list [5, 7, 8]. PICE diagnoses not appearing on these lists were classified before analyzing the data according to expert opinion (CV, JL) [35]. The list of the PICE database diagnoses grouped as a CCC and NCCC is described in Table 8 and Table 9 (online only).

Socio-economic status of the family was obtained by coupling the four digits of the postal code to the socio-economic status of the neighborhood in 2006 (The Netherlands Institute for Social Research) and grouped into three categories [32].

### Data analysis

Normal distribution of continuous variables was tested using sampling distributions and skewness and kurtosis tests. Not normally distributed data were reported by median and inter-quartile range (IQR). Non-parametric tests (Mann-Whitney *U*) were used for the analyses of not normally distributed data. For categorical variables, Fisher’s exact test or the chi-square test was used (software: IBM SPSS Statistics 22).

### Reliability study

To assess the reliability of the record review process, a random sample of nine records (20%) was reviewed by a second investigator.

## Results

### Respondent characteristics

**Fig. 1:**
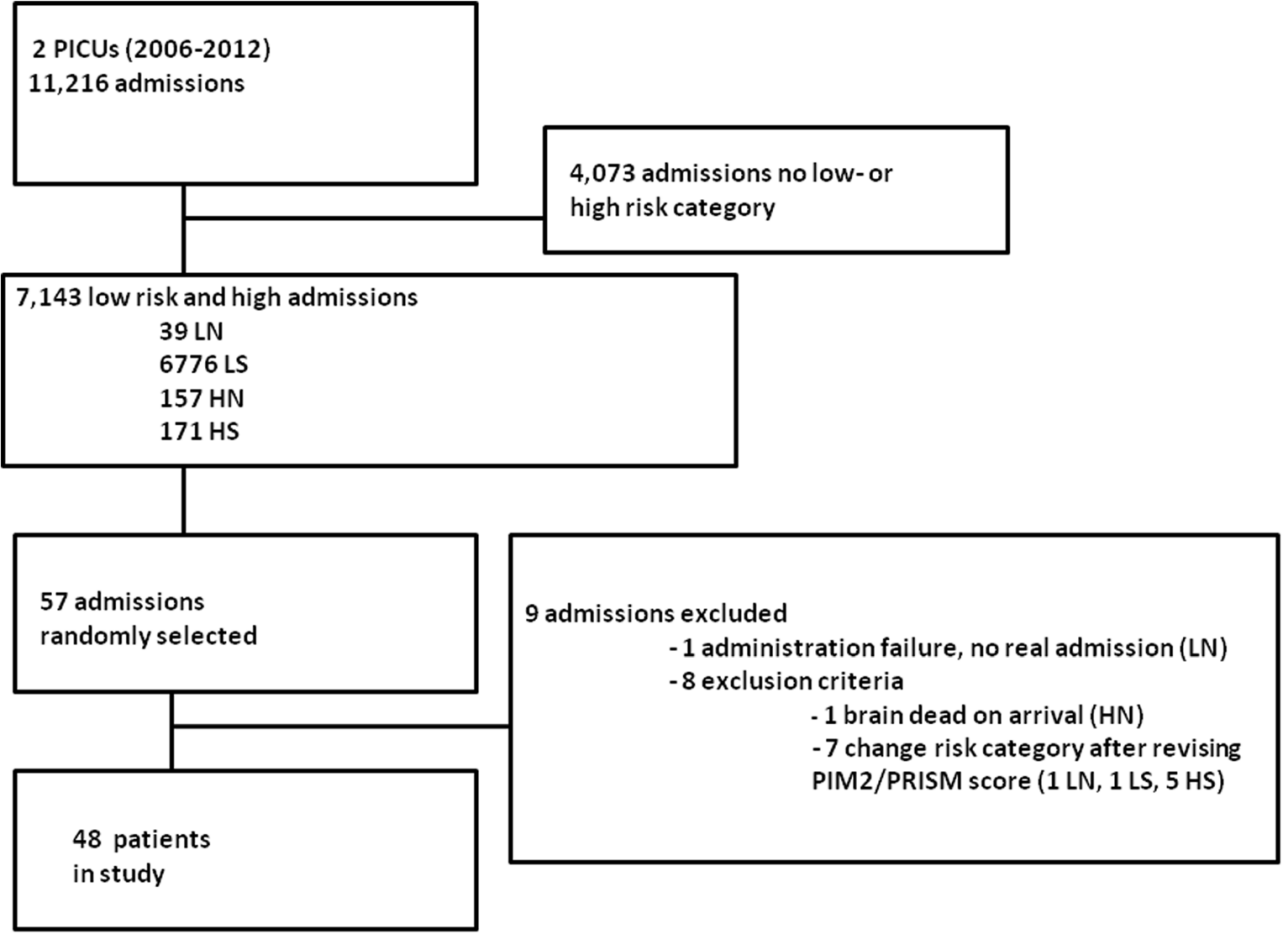
Flowchart of the study population. LN low-risk non-survivor, LS low-risk survivor, HN high-risk non-survivor, HS high-risk survivor, PIM2 Pediatric Index of Mortality score, PRISM Pediatric Risk of Mortality

A total of 48 patients were randomly selected. Nine admissions were excluded, and therefore, nine new admissions were chosen as described (Fig. 1, flowchart).

Patient characteristics are listed in Table 1. The four groups were different on admission characteristics, mortality risk scores, presence of CCCs, and outcome characteristics like length of stay. The LN group had more medical admissions and higher PRISM mortality risk compared to the LS group. The PIM2 mortality risks between LN and LS were comparable. LN patients were more often mechanically ventilated; had more ventilator days, more central venous catheters, and more central venous catheter days; and had a longer length of stay compared to LS patients.

**Table 1:** Patient characteristics

Characteristic	LN	LS	HN	HS
Patients in each subgroup	12	12	12	12
Gender: male	6	6	6	6
Age group				
• 1–28 days	1	1	1	1
• 29–365 days	4	4	4	4
• 1–4 years	0	0	0	0
• 5–17 years	7	7	7	7
Age: median [IQR] (years)	9.5 [0–12.8]	7.5 [0–13.0]	5.0 [0–13.3]	5.5 [0–11.3]
Weight: median [IQR] (kg)	32.5 [3.9–53.5]	14.9 [3.1–44.8]	20.0 [7.0–50.0]	22.0 [5.5–37.0]
Socio-economic status				
• Low	3	3	2	3
• Intermediate	5	8	8	8
• High	3	1	1	1
• Unknown	1	0	1	0
Non-elective admission	10	7^d,f^	12	12
Medical admission	12^aa,c^	6	8	10
CPR or brain herniation as the cause for PICU admission	0	0	9^b^	3
Off-hours admission	6	4	6	7
Chronic condition				
• CCC	9^cc^	7	3^b^	6
• NCCC	2	1	0	3
• None	1	4	9	3
Recalibrated PRISM mortality risk, median [IQR] (%)	0.9 [0.7–1.4]^a,ccc,eee^	0.6 [0.5–0.8]^ddd,fff^	77.0 [21.4–87.4]	43.6 [35.3–60.5]
Recalibrated PIM2 mortality risk, median [IQR] (%)	1.3 [0.8–6.1]^ccc,e^	1.3 [1.0–2.2]^d,fff^	56.1 [21.8–83.4]^b^	14 [14–46]
Mechanical ventilation	11^aa^	4^dd,ff^	12	12
Ventilator days, median [IQR]	6.5 [2.5–30.8]^aaa^	0 [0–1.8]^ddd,ff^	2.5 [1.0–9.3]	6.5 [4.3–11.5]
Central venous catheter	10^a^	5^ff^	11	9
Central venous catheter days, median [IQR]	4.5 [1.3–14.3]^aa^	0 [0–2]^dd,ff^	2.5 [1–17.5]	6.5 [1–11.8]
Extracorporal life support	2*	0	1	3
Length of stay, median [IQR] (days)	16 [5.5–32.8]^aa,c,e^	2 [2–2.8]^dd^	2.5 [1–9.3]^b^	11 [6.3–13]
Mode of death (*n* = 24)		Not applicable		Not applicable
- Brain death	0^c^		6	
- Maximal treatment including CPR	1		0	
- Maximal treatment without CPR	2		1	
- Limiting or withdrawal of therapy	9		5	

All numbers are expressed as the number of patients unless specified otherwise

*LN* low-risk non-survivors, *LS* low-risk survivors, *HN* high-risk non-survivors, *HS* high-risk survivors

^*^Two patients in LN with extracorporal life support (ECLS): one patient, a neonate with a very complex congenital cardiac disorder including pulmonary atresia and total abnormal pulmonary venous return, was admitted preoperatively for cardiac surgery and needed ECLS after surgery but did not survive. The mortality risk in this patient was—according to the PIM2/PRISM criteria—measured before surgery and was low. Another patient, admitted with severe asthma, was resuscitated during PICU stay (day 2) and supported by ECLS after resuscitation but died of cerebral post-anoxic complications

^a^*p* < 0.05, ^aa^*p* < 0.01, and ^aaa^*p* < 0.001, LN compared with LS; ^b^*p* < 0.05, HN compared with HS; ^c^*p* < 0.05, ^cc^*p* < 0.01, and ^ccc^*p* < 0.001, LN compared with HN; ^d^*p* < 0.05, ^dd^*p* < 0.01, and ^ddd^*p* < 0.001, LS compared with group HS; ^e^*p* < 0.05, and ^eee^*p* < 0.001, LN compared with group HS; ^f^*p* < 0.05, ^ff^*p* < 0.01, and ^fff^*p* < 0.001, LS compared with group HN

In the LN group, most patients had a CCC (not resulting in a higher PIM2 or PRISM score) in contrast to the HN, where CCCs occurred in a minority of patients. In the HN, cardiopulmonary resuscitation was a frequent reason for admission, often resulting in brain death as the cause of death. In the majority of the LN, patients died after limiting therapeutic options. The length of stay in the LN was much longer compared to the HN and also longer compared to the HS.

### Adverse events

The occurrence of AEs in the LN group was significantly higher compared to that in the LS and HN groups (Table 2). Eighty-three percent of the LN patients suffered from at least one AE. Twenty-five AEs occurred in the LN group. The AE rate (AE per PICU day) in the LN group was significantly higher compared to that in the LS and HN groups (median 0.12 AE/PICU day).

**Table 2:** Adverse events

Outcome measure	LN	LS	HN	HS
Patients with ≥ 1 AE(/*n*)	10/12^aaa,cc^	1/12^dd^	2/12^b^	7/12
AE PICU/PICU day, median [IQR]	0.12 [0.07–0.29]^aaa,cc^	0 [0–0]^dd^	0 [0–0]^b^	0.03 [0.0–0.17]
Number of AEs, total	25	2	8	10
Number of AEs/patient, median [IQR]	2 [1–3.8]	0 [0–0]	0 [0–0]	1 [0–1]

Only the primary outcome (patients with greater than or equal to one AE) and AE rate were tested

*LN* low-risk non-survivors, *LS* low-risk survivors, *HN* high-risk non-survivors, *HS* high-risk survivors, *AE* adverse event, *PICU* pediatric intensive care unit, *AE PICU/PICU day* the number of AEs per patient day

^aaa^*p* < 0.001, LN compared with LS; ^b^*p* < 0.05, HN compared with HS; ^cc^*p* < 0.01, LN compared with HN; ^dd^*p* < 0.01, LS compared with group HS, LN compared with group HS; ^f^*p* < 0.05, ^ff^*p* < 0.01, and ^fff^*p* < 0.001, LS compared with group HN

In Table 3, preventability, severity, and classification of all identified AEs are shown. In the LN group, eight preventable AEs occurred. In five of these preventable AEs, the severity was high (grade G-I). Two patients, in both the LN groups, died after a preventable AE. Looking at all 15 preventable AEs found among all subgroups in this study, most preventable AEs were related to problems in clinical monitoring (*n* = 5), infection control (*n* = 5), and diagnosis (*n* = 2). Detailed information about all patients with AEs including description, timing, severity, and preventability of the AEs is shown in Table 10 (online only). The day on which the AE occurred varied from day 0 (preceding the PICU admission) to the last days of the PICU stay.

**Table 3:** Preventability, severity, and classification of adverse events

Group	No AEs	Preventability	Severity	Classification
LN	25	8 preventable AEs	I = 2	Infection control = 1
				Clinical monitoring = 1
			G–H = 3	Drug or fluid related = 1
				Diagnosis = 2
			E–F = 3	Infection control = 2
				Clinical monitoring = 1
		17 non-preventable AEs	I = 4	Other = 3
				Drug or fluid related = 1
			G–H = 5	Other = 4
				Drug or fluid related = 1
			E–F = 8	Infection control = 4
				Other = 3
				Technical = 1
LS	2	2 preventable AEs	H = 2	Infection control = 1
				Drug or fluid related = 1
HN	8	2 preventable AEs	G–H = 1	Clinical monitoring = 1
			E–F = 1	Infection control = 1
		6 non-preventable AEs	I = 1	ECLS = 1
			G–H = 1	ECLS = 1
			E–F = 4	ECLS = 1
				Other = 3
HS	10	3 preventable AEs	G–H = 1	ECLS = 1
			E–F = 2	ECLS = 1
				Clinical monitoring = 1
		7 non-preventable AEs	G–H = 5	Clinical monitoring = 1
				ECLS = 1
				Other = 3
			E–F = 2	ECLS = 1
				Technical = 1
Total	45	15 preventable		Clinical monitoring = 4
		30 unpreventable		Diagnosis = 2
				Drug or fluid related = 2
				ECLS = 2
				Infection control = 5
				Clinical monitoring = 1
				Drug or fluid related = 2
				Technical = 2
				ECLS = 5
				Infection control = 4
				Other = 16

Severity categories: E = contributed to or resulted in temporary harm to the patient and required intervention, F = contributed to or resulted in temporary harm to the patients and required initial or prolonged hospitalization, G = contributed to or resulted in permanent patient harm, H = required intervention to sustain life, I = contributed to or resulted in the patient’s death

*LN* low-risk non-survivors, *LS* low-risk survivors, *HN* high-risk non-survivors, *HS* high-risk survivors, *AE* adverse event, *ECLS* extracorporal life support

### Inter-observer agreement

Nine patient records were reviewed by the second investigator. Inter-observer agreement was 8/9 (89%).

## Discussion

### Major findings

In this exploratory study, AEs occurred in 83% of the LN. The occurrence of AEs and AE rate in these LN patients were significantly higher compared to those in LS patients and also higher compared to those in HN patients. A substantial part of the AEs in the LN group was preventable and had severe consequences, including two LN patients who died after a preventable AE. Screening patients with a low mortality risk is a valuable tool to discover problems in the quality of care and might reduce preventable death by implementing targeted quality improvement measures.

A possible explanation for the higher occurrence of AEs in the LN group might be that “low-risk” as defined by a calculated low mortality risk does not always reflect a true low risk of dying. Mortality risk scores such as PIM2 or PRISM scores perform reasonably well for the PICU population in general with an AUC between 0.83 and 0.90 but not for each individual [37]. Many patients in the LN group are sicker than they appear based on the PIM2 or PRISM score. Misclassifications do occur. For example, seven LN patients were admitted to the PICU with major comorbidity such as hemato-oncology patients and patients with complex congenital heart disorder. These low-risk patients with a CCC are often at high risk for AEs [35]. Patients with congenital heart disorders are sometimes admitted preoperatively to the PICU. Mortality risk scores can be obtained before surgery and do not measure true postoperative risk. New PRISM methods like PRISM IV might reflect mortality risk better in these patients because the risk score is measured after surgery [26]. However, severe and preventable AEs did occur in patients with and without a CCC, so to our opinion, this is not the only explanation.

Comparing the AE rate from this study with other studies is difficult because in this exploratory study, we did not include the general PICU population but focused on the low- and high-risk groups. A single PICU study on patient safety factors in 47 PICU non-survivors found that 36% of non-survivors suffered at least one AE of category I and 60% suffered a “critical incident” [19]. These results cannot be compared with our study not only because of different population characteristics but also due to different outcome measures. The “critical incidents” used in the study of Monroe could either be AEs or medical errors not causing harm (categories B–D), a category which is too wide in our opinion [21].

From the viewpoint of quality improvement, preventable AEs are the most interesting. Looking at the nature of the 15 preventable AEs found in this study, problems in clinical monitoring (*n* = 5), infection control (*n* = 5), and diagnosis (*n* = 2) were most prevalent. For example, a pediatric early warning system might lead to timely recognition of deterioration and thus lead to lower mortality [11]. During the study period, pediatric early warning systems and sepsis bundles were implemented in the participating hospitals, but the effectiveness could not be systematically examined yet.

The length of stay in the LN group was significantly longer compared to that in all other groups. A longer duration of stay may be the consequence of the AEs or might have contributed to an increased chance for AEs, and this cannot be estimated from this retrospective study.

### Limitations

Our study has several limitations. First, children in the age group of 1–4 years were not present in the randomly chosen LN group and therefore not in the other groups, possibly giving rise to bias. Second, a relatively high number of admissions were excluded from the study. The decision to exclude patients was made on predefined criteria. Remarkably, in seven patients, the PIM2/PRISM score turned out to be false after verifying with the data from the medical record. This should encourage better surveillance of the database. Third, poor quality of the information in patient records might lead to underestimation of the number of AEs. The assessment of AEs with a trigger tool method depends on the presence of data in the medical record. However, in a patient record review study in Dutch hospitals, poor quality of the information present in the medical record was associated with higher rates of AEs [40]. Another weakness of all retrospective studies is hindsight bias [9, 39]. Knowledge of the final outcome may have influenced judgment on severity and preventability. This could lead to an overestimation of preventable severe AEs as judged by the investigators. Finally, the mortality prediction models do not perform perfectly. However, we found that both in real LN and in LN with a CCC, severe AEs and AEs contributing to death occur.

## Conclusion

This exploratory study shows that AEs do occur in PICU low-risk non-survivors. The occurrence of AEs in low-risk non-survivors was higher compared to that in low-risk survivors and high-risk non-survivors. Some AEs were severe and preventable and contributed to morbidity and mortality. The exact scale and nature of this safety problem should be analyzed in a larger multi-center study.

## Electronic supplementary material

ESM 1 (DOCX 15 kb)

ESM 2 (DOCX 12 kb)

ESM 3 (DOCX 13 kb)

ESM 4 (DOCX 13 kb)

ESM 5 (DOCX 19 kb)

ESM 6 (DOCX 16 kb)

ESM 7 (DOCX 20 kb)

## Abbreviations

AE: Adverse event
CCC: Complex chronic condition
ECLS: Extracorporal life support
HN: High-risk non-survivor
HS: High-risk survivor
IQR: Inter-quartile range
LN: Low-risk non-survivor
LS: Low-risk survivor
NCCC: Non-complex chronic condition
NCC MERP: National Coordinating Council for Medication Error Reporting and Prevention
PICU: Pediatric intensive care unit
PIM: Pediatric Index of Mortality
PRISM: Pediatric Risk of Mortality
