# Supplementary material for: The occurrence of adverse events in low-risk non-survivors in pediatric intensive care patients: an exploratory study
Source: Eur J Pediatr. 2018 Jun 26;177(9):1351–8. doi: 10.1007/s00431-018-3194-y (PMC6096770; doi:10.1007/s00431-018-3194-y)
Supplement: Supplementary file 1 — (DOCX 15 kb) [file 431_2018_3194_MOESM1_ESM.docx]

**Table 4, online only: Triggers used to identify adverse events**

(modification of triggertool used by Agarwal) ^[15]^

| **No** | **Trigger** | **Examples / Potential AEs** |
| --- | --- | --- |
| 1 | Cardiac or respiratory arrest | Resuscitation, defibrillation, cardioversion, emergency intubation, administration of epinephrine |
| 2 | Accidental extubation |  |
| 3 | Pulmonary | Pneumothorax, chylothorax , aspiration pneumonia |
| 4 | Neurology | CNS bleed, CNS ischemia/infarction |
| 5 | Infectious disease | Infection of any kind occurring > 3 days after admission |
| 6 | Subcutaneous infusion | Need for hyaluronidase infusion |
| 7 | Decubitus ulcer (pressure sores) |  |
| 8 | Readmission < 48 hours |  |
| 9 | Central catheter | Central catheter clot, inadvertent catheter removal, bleeding from central catheter, change of ECLS system |
| 10 | Trachea | Post extubation stridor, racemic epinephrine administration |
| 11 | Dislocation endotracheal tube | Order to pull back or push ETT or chest X ray with tube > 0.5 cm to (un)deep, not direct after intubation/ ETT mal-positioning requiring reposition |
| 12 | Oversedation | COMFORT-B score < 11 during 24 hours |
| 13 | Allergy | Allergic reaction, treatment with clemastine, allergic rash |
| 14 | Pain,undersedation | Uncontrolled pain, undersedation (two times COMFORT-B score > 17 and/or NRS > 4 within one hour) |
| 15 | Hypo-/ hyperglycaemia | Insulin treatment, glucose <4 or > 8 mmol/l in children, glucose <2.7 or >8 mmol/l in neonates |
| 16 | Withdrawal symptoms | Notification of withdrawal symptoms in medical record, use of medications (like methadon, lorazepam, clonidine orally), SOS score > 4 twice. |
| 17 | Delirium | Notification of delirium in medical record, use of medications like haloperidol, risperidon, combinations of different scores (CAP-D score, pCAM-ICU score, SOS-PD score) |
| 18 | Thrombosis | Deep vein thrombosis |
| 19 | Other | Other incidents: unplanned return to surgery, problems with foley catheter, problems with epidural catheter, falling incidents, diagnostic delay |

**Legend table 4**

CNS = central nervous system, ECLS = extra corporal life support, ETT = endotracheal tube, COMFORT-B score= COMFORT behavioral score (sedation score), NRS =Numerical Rating Scale (pain score), SOS-score = Sophia Observation withdrawal Score, CAP-D score = Cornell Assessment of the Pediatric Delirium, pCAM-ICU = pediatric Confusion Assessment Method for the Intensive Care Unit, SOS-PD score = Sophia Observation withdrawal Score – Pediatric Delirium
