# Supplementary material for: The occurrence of adverse events in low-risk non-survivors in pediatric intensive care patients: an exploratory study
Source: Eur J Pediatr. 2018 Jun 26;177(9):1351–8. doi: 10.1007/s00431-018-3194-y (PMC6096770; doi:10.1007/s00431-018-3194-y)
Supplement: Supplementary file 2 — (DOCX 12 kb) [file 431_2018_3194_MOESM2_ESM.docx]

**Table 5, online only: Harm classification using the NCC MERP criteria** [30]

| **Category** | **Definition** | **Error? Harm?** |
| --- | --- | --- |
|  |  |  |
| **A** | Circumstances or events that have the capacity to cause error | No error |
| **B** | An error occurred but the error did not reach the patient | Error, no harm |
| **C** | An error occurred that reached the patient but did not cause patient harm | Error, no harm |
| **D** | An error occurred that reached the patient and required monitoring to confirm that it resulted in no harm to the patient and/or required intervention to preclude harm | Error, no harm |
|  |  |  |
| **E** | Contributed to or resulted in temporary harm to the patient and required intervention | Harm |
| **F** | Contributed to or resulted in temporary harm to the patients and required initial or prolonged hospitalization | Harm |
| **G** | Contributed to or resulted in permanent patient harm | Harm |
| **H** | Required intervention to sustain life | Harm |
| **I** | Contributed to or resulted in the patient’s death | Death |

**Legend table 5:**

NCC MERP: National Coordination Council for Medication Error Reporting and Prevention

Categories E-I = Adverse event
