# Supplementary material for: The occurrence of adverse events in low-risk non-survivors in pediatric intensive care patients: an exploratory study
Source: Eur J Pediatr. 2018 Jun 26;177(9):1351–8. doi: 10.1007/s00431-018-3194-y (PMC6096770; doi:10.1007/s00431-018-3194-y)
Supplement: Supplementary file 3 — (DOCX 13 kb) [file 431_2018_3194_MOESM3_ESM.docx]

**Table 6 : Preventability of AEs**[5]

| **Category** | **Definition** |
| --- | --- |
| **1** | (Virtually) no evidence for preventability |
| **2** | Slight to modest evidence of preventability |
| **3** | Preventability not quite likely (less than 50/50, but ‘close call’) |
| **4** | Preventability more than likely (more than 50/50, but ‘close call’) |
| **5** | Strong evidence of preventability |
| **6** | (Virtually) certain evidence of preventability |

**Legend table 6:**

AE = adverse event

AEs with a preventability score of 4 to 6 were defined as preventable AEs.
