# Supplementary material for: The occurrence of adverse events in low-risk non-survivors in pediatric intensive care patients: an exploratory study
Source: Eur J Pediatr. 2018 Jun 26;177(9):1351–8. doi: 10.1007/s00431-018-3194-y (PMC6096770; doi:10.1007/s00431-018-3194-y)
Supplement: Supplementary file 4 — (DOCX 13 kb) [file 431_2018_3194_MOESM4_ESM.docx]

**Table 7: Classification of adverse events**

**Modification of classification made by Hogan** [31]

| **Type of problem** | **Definition** |
| --- | --- |
| **Clinical monitoring** | Failure to act upon results of tests or clinical findings, set up monitoring systems or respond to such systems or increase intensity of care when required |
| **Diagnosis** | Missed, delayed or inappropriate diagnosis as a result of failure to perform an adequate assessment of patient’s overall condition including appropriate tests or lack of focused assessment when required |
| **Drug or fluid related** | Side effects, inappropriate use, failure to give prophylactic care, anaphylaxis, etc |
| **Technical problems** | Related to a device, an operation or procedure wheter on ward, in a diagnostic stuite or in theatre and including inappropriate or unnecessary procedures (*other than technical problems related to ECLS)* |
| **ECLS** | Problems related to ECLS including technical problems, hemorrhage |
| **Infection related** | Healthcare associated infections including infections from indwelling device |
| **Resuscitation** | Problems in resuscitation including cardiopulmonary resuscitation such as delay in beginning resuscitation, problems related to resuscitation technique, resuscitation medication/fluids, resuscitation equipment |
| **Other** | Any other problem not fitting categories above |

**Legend table 7:**

ECLS = extracorporal life support
