# Supplementary material for: The occurrence of adverse events in low-risk non-survivors in pediatric intensive care patients: an exploratory study
Source: Eur J Pediatr. 2018 Jun 26;177(9):1351–8. doi: 10.1007/s00431-018-3194-y (PMC6096770; doi:10.1007/s00431-018-3194-y)
Supplement: Supplementary file 5 — (DOCX 19 kb) [file 431_2018_3194_MOESM5_ESM.docx]

**Table 8: List of diagnoses classified as complex chronic conditions (CCC)**

**(Modification of Feudtner’s list)** [2, 33-35]

| **Complex chronic conditions** | |
| --- | --- |
| **Subgroup** | **Diagnoses from the PICE database** |
| **Cardiovascular** | Absent pulmonary valve syndrome* Anomaly of the coronary artery Arterial switch* Atrioventricular septal defect Cardiomyopathy Cavopulmonary shunt* Cor triatriatum Double outlet right ventricle Ebstein’s anomaly Fontan procedure* Hypoplastic left heart syndrome Hypoplastic left ventricle* Hypoplastic or interrupted aortic arch* Hypoplastic right ventricle* Levo transposition of the great arteries Mitral valve stenosis  Monoventricle Norwood procedure – step 1* Pacemaker insertion/revision* Portal hypertension* Pulmonary atresia or stenosis Pulmonary artery banding* Reconstruction of aortic arch* Reconstruction of left ventricular outflow* Reconstruction of right ventricular outflow* Restoration of atrioventricular septumdefect* Repair of plastic pulmonary artery* Repair or replacement of conduit* Repair of tetralogy of Fallot* Right ventricular outflow tract obstruction* Senning procedure* Supraventricular arrhythmia Surgery of pulmonary collateral arteries* Systemic to pulmonary shunt procedure* Tetralogy of Fallot Total abnormal pulmonary venous return Transplantation of heart Transplantation of heart and lung Transplantation of heart and lung – state after procedure Transposition of the great arteries Tricuspid atresia or stenosis Truncus arteriosus Vasculitis* Ventricular arrhythmia |
| **Respiratory** | Bronchiectasis  Central apnoea* Choanal atresia or stenosis* Chronic lung disease* Congenital lung disease Cystic fibrosis Infant respiratory distress syndrome* Laryngomalacia Malacia trachea or bronchus Massa mediastinum* Pulmonary edema Pulmonary hypoplasia Pulmonary insufficiency* Reconstuction of larynx* Subglottic stenosis Tracheostomy* Trachea or bronchus stenosis Transplantation of lung Transplantation of lung – state after procedure Vocal cord paralysis* |
| **Hematological** | Coagulation defects Hematologic disease* |
| **Endocrinological** | Congenital metabolism disorder Diabetes (comorbidity)* Diabetes inspididus Diabetes mellitus with ketoacidosis Diabetes mellitus without ketoacidosis Endocrine disorder Kasaï procedure* |
| **Gastrointestinal** | Biliary atresia Colitis Congenital diaphragmatic hernia Gastroschisis or exomphalus Hirschsprung’s disease* Liver disease – other* Oesophageal atresia Repair of esophageal atresia* Repair of esophageal fistel* Repair of total anomalous pulmonary venous return* Short bowel syndrome* Transplantation of kidney Transplantation of liver Transplanation of liver – state after procedure Transplantation of small intestine Varices of oesophagus or stomach* |
| **Immunological** | Congenital immunodeficiency Graft versus host disease Neutropenia* Pancytopenia* Pheochromocytoma* |
| **Neuromuscular** | Acute disseminated encephalomyelitis* Arnold-Chairi malformation Brain arteriovenous malformation* Brain tumour Central nervous system shunt dysfunction or infection* Cerebral aneurism Cerebral cyst Cerebral infarction* Chronic traumatic encephalopathy  Congenital brain disease* Convulsions* Craniotomy – fossa anterior* Epilepsy (comorbidity) Hydrocephalus Insertion of revision of central nervous system shunt* Lobectomy or hemispherectomy* Meningomyelocele or spina bifida  Muscular dystrophy Myastenia gravis Myelum – impairment* Myopathy Repair of myelomeningocele* Static encephalopathy |
| **Oncological** | Cystic hygroma Leukemia or lymphoma Malignant solid organ neoplasm Transplantation of bone marrow Transplantation of bone marrow – state after procedure |
| **Renal** | Chronic kidney failure Hydronephrosis* Nephrotic or nephritic syndrome* Transplantation of kidney – state after procedure |
| **Endocrinal** | Syndrome of inappropriate antidiuretic hormone secretion* |
| **Genetic** | Chromosome abnormality Craniosynotosis* DiGeorge syndrome Down syndrome Pierre Robin syndrome* |
| **Urological** | Repair of exstrophia vesicae* |
| **Miscellaneous** | Syndrome or malformation* |

**Legend table 8**

* Diagnoses that were not on the original Feudtner’s list (as CCC)
