# Supplementary material for: The occurrence of adverse events in low-risk non-survivors in pediatric intensive care patients: an exploratory study
Source: Eur J Pediatr. 2018 Jun 26;177(9):1351–8. doi: 10.1007/s00431-018-3194-y (PMC6096770; doi:10.1007/s00431-018-3194-y)
Supplement: Supplementary file 6 — (DOCX 16 kb) [file 431_2018_3194_MOESM6_ESM.docx]

**Table 9: List of diagnoses classified as non-complex chronic conditions (NCCC)**

**(modification of Feudtner’s list)**[33-35]

| **Non-complex chronic conditions** | |
| --- | --- |
| **Subgroup** | **Diagnoses from the PICE database** |
| **Cardiovascular** | Aorta insufficiency Aorta stenosis Atrial septal defect Aortopulmonary window* Arteriovenous malformation* Acquired cardiovascular disorder – other* Coartectomy* Coarctatio aortae Closed valvotomy* Closed heart surgery – other* Congenital cardiovascular disorder- other Ductus arteriosus Left ventricle outflow tract obstruction* Mitral insufficiency Myocardal infarction or ischemia* Open valvotomy* Open heart surgery – other* Pulmonary hypertension* Past heart surgery* Repair of atrial septal defect* Repair of ventricular septal defect* Repair of coronary artery* Repair of ductus arteriosus* Repair or replacement of valve* Systemic hypertension Tricuspidal insufficiency Ventricular septal defect |
| **Respiratory** | Asthma Chylous effusion* Obstructive sleep apnoea syndrome Pneumectomy or lobectomy* |
| **Gastrointestinal** | Repair of gastroschisis or exomphalos* |
| **Neuromuscular** | Guillain Barré syndrome* Neuropathy Neurosurgery – other* |
| **Oncological** | Cardiac tumour* Non-malignant solid organ neoplasm Resection of abdominal tumour* Resection of cardiac tumour * Resection of thoracic tumour*  Subglottic hemangioma |
| **Genetic** | Repair of cheiloschisis* Repair of palatoschisis* |
| **Miscellaneous** | Scoliosis |

**Legend table 9:**

NCCC = non complex chronic condition

* Diagnoses that were not mentioned on the original Feudtner’s list (as NCCC)
