# Supplementary material for: The occurrence of adverse events in low-risk non-survivors in pediatric intensive care patients: an exploratory study
Source: Eur J Pediatr. 2018 Jun 26;177(9):1351–8. doi: 10.1007/s00431-018-3194-y (PMC6096770; doi:10.1007/s00431-018-3194-y)
Supplement: Supplementary file 7 — (DOCX 20 kb) [file 431_2018_3194_MOESM7_ESM.docx]

**Table 10 : Detailed description of patients with AEs**

(AEs occurred in 20/48 patients)

| **Group/patient** | **Age**  **(yr)** | **Diagnosis** | **CCC** | **No. AE** | **LoS** | **Timing AE (day)** | **Location AE**  **(pre PICU /PICU)** | **Description of AE** | **Severity** | **Preventability (x/6)** |
| --- | --- | --- | --- | --- | --- | --- | --- | --- | --- | --- |
| **LN** |  |  |  |  |  |  |  |  |  |  |
| 1 | 10 | Acute myeloid leukemia, neutropenic enterocolitis | CCC | 1 | 7 | 6 | PICU | Abdominal compartment syndrome | Cat H | 1 |
| 2 | 12 | Acute myeloid leukemia, hyperleuco-cytosis | CCC | 1 | 3 | 2 | PICU | Fluid overload | Cat H | 4 |
| 3 | 9 | Trauma, pelvic fracture | no CCC | 3 | 1 | 0  0  1 | 2 pre PICU  1 PICU | Missed diagnosis gut perforation  Fat embolism  Tension pneumothorax (found post mortem) | Cat H  Cat I  Cat I | 4  3  1 |
| 4 | 13 | Morbus Steinert, pneumonia | NCCC | 3 | 32 | 23  26  30 | PICU | Sudden resuscitation  Urinary tract infection  Post anoxic encephalopathy | Cat G  Cat E  Cat I | 1  2  2 |
| 5 | 15 | Epilepsy, status epilepticus | CCC | 4 | 36 | 3  21  22  33 | PICU | CLABSI  CLABSI  Urinary tract infection  Deep vein thrombosis (despite prophylaxis) | Cat E  Cat E  Cat E  Cat E | 5  5  2  1 |
| 6 | 0 | Premature, short bowel after necrotising enterocolitis, cholestasis | CCC | 1 | 16 | 16 | PICU | CLABSI | Cat I | 4 |
| 7 | 0 | Giant omphalocele, pulmonary hypertension | CCC | 1 | 10 | 5 | PICU | Extravasation injury | Cat E | 4 |
| 8 | 17 | Juvenile chronic arthritis, hemophago-cytic lympho-histiocytosis | CCC | 4 | 16 | 0  1  11  14 | 1 pre PICU  3 PICU | Gastric perforation during steroids (despite prophylaxis)  Delay in diagnosis of gastric perforation  Pneumothorax  Cerebral Hemorrhage | Cat H  Cat H  Cat E  Cat I | 1  4  1  2 |
| 9 | 11 | Status asthmaticus | NCCC | 3 | 5 | 2  2  3 | PICU | Resuscitation during spontaneous ventilation in PICU  Hypoglycaemia  Pneumothorax (on ECLS) | Cat I  Cat E  Cat E | 4  1  1 |
| 10 | 0 | Hypoplastic right ventricle, total abnormal venous return | CCC | 4 | 33 | 20  20  26  28 | PICU | Resuscitation - SVT  Resuscitation  CLABSI (on ECLS)  CLABSI | Cat H  Cat H  Cat E  Cat E | 1  1  2  2 |
| **LS** |  |  |  |  |  |  |  |  |  |  |
| 11 | 12 | Marfan syndrome, Bental procedure, hematothorax | CCC | 2 | 2 | 0  0 | 2 pre PICU | Infected pericardial effusion  Hematothorax, no antagonising coumarines before inserting central venous line | Cat H  Cat H | 4  5 |
| **HN** |  |  |  |  |  |  |  |  |  |  |
| 12 | 11 | Trauma, cardiac arrest at trauma site | no CCC | 1 | 1 | 0 | pre PICU | Hypoxia and hypotension during transport to PICU | Cat H | 4 |
| 13 | 14 | Necrotizing pneumonia (influenza, staphylococcus aureus ), transfer from another PICU for ECLS | no CCC | 7 | 21 | 5  9  9  11  15  18  21 | PICU | Small cerebral hemorrhage (on ECLS)  Pneumothorax  Hematothorax after drainage of pneumothorax on ECLS  Pneumothorax  Pneumothorax  CLABSI  Cerebral hemorrhage (on ECLS) | Cat E  Cat E  Cat H  Cat E  Cat E  Cat E  Cat I | 2  1  2  1  1  4  2 |
| **HS** |  |  |  |  |  |  |  |  |  |  |
| 14 | 9 | Cerebral herniation, hydrocephalus, neurofibromatosis | CCC | 1 | 24 | 13 | PICU | Decubitus | Cat E | 4 |
| 15 | 0 | Meconium aspiration syndrome, transfer for ECLS | no CCC | 3 | 12 | 1  3  5 | PICU | Resuscitation before ECLS was started  Replacement ECLS canule (wrong canule placed)  Cerebral hemorrhage on ECLS | Cat H  Cat E  Cat G | 1  6  2 |
| 16 | 0 | Urgent laparotomy, bleeding from large abdominal tumor | CCC | 1 | 13 | 1 | PICU | Abdominal compartment syndrome / resuscitation | Cat H | 2 |
| 17 | 0 | Trisomie 21, meningo-encephalitis, septic shock | CCC | 2 | 8 | 1  1 | PICU | Subdural empyema and cerebral hemorrhage  Necrosis of digits | Cat G  Cat G | 1  1 |
| 18 | 12 | Out of hospital resuscitation, aspiration, ARDS, transfer from another PICU for ECLS | no CCC | 1 | 5 | 2 | PICU | Hemorrhage around ECLS canula in left groin | Cat E | 1 |
| 19 | 12 | Status asthmaticus | NCCC | 1 | 11 | 3 | PICU | Ischaemia leg, on veno-arterial ECLS | Cat G | 4 |
| 20 | 5 | Juvenile myelomono-cytic leukemia, bone marrow transplant, graft versus host, short bowel, seizures | CCC | 1 | 41 | 21 | PICU | Obstruction of CVL | Cat E | 1 |

`

**Legend table 10**

**Abbreviations**: LN = low-risk non-survivors, LS = low-risk survivors, HN = high-risk non-survivors, HS = high-risk survivors, CCC= complex chronic condition, NCCC = non-complex chronic condition, no CCC= no (non-) complex chronic condition, AE = adverse event, No.AE = number of AEs in this patient, LoS = length of stay (days), PICU = pediatric intensive care unit, CLABSI = central line-associated blood stream infection, ECLS = extracorporal life support, CVL = central venous line

**Timing AE** (day) = day op PICU admission when AE occurred, if an AE was preceding the PICU admission, it was scored as day 0

**Severity categories**: Cat(egory) E = Contributed to or resulted in temporary harm to the patient and required intervention, Cat F = Contributed to or resulted in temporary harm to the patients and required initial or prolonged hospitalization, Cat G = Contributed to or resulted in permanent patient harm, Cat H = Required intervention to sustain life, Cat I = Contributed to or resulted in the patient’s death
